# 582. Comparing Broad-range PCR Testing and The Biofire® FilmArray® Pneumonia (PN) Panel in the Diagnosis of Bacterial Pneumonia

**DOI:** 10.1093/ofid/ofad500.651

**Published:** 2023-11-27

**Authors:** Haseeba Khan, Kirsten Prabhudas-Strycker, Matthew Samaro, Kagan A Mellencamp, Michael Goings, LaKeisha Boyd, Jack Schneider, Christopher L Emery

**Affiliations:** Indiana University, Indianapolis, Indiana; Indiana University, Indianapolis, Indiana; Indiana University, Indianapolis, Indiana; Indiana University, Indianapolis, Indiana; Indiana University, Indianapolis, Indiana; Indiana University, Indianapolis, Indiana; Indiana University School of Medicine, Indianapolis, Indiana; Indiana University School of Medicine, Indianapolis, Indiana

## Abstract

**Background:**

Given the low sensitivity of conventional microbial isolation methods for identifying respiratory pathogens in bacterial pneumonia, target-specific syndromic multiplex real-time PCR panels have been used in conjunction with culture methods to improve diagnostic yield. Additionally, broad-range polymerase chain reaction (BR-PCR) targeting bacterial 16s rRNA conserved region has shown higher sensitivity with certain specimen types, so we sought to evaluate the clinical performance of BR-PCR performed on bronchoalveolar lavage (BAL) specimens in comparison to The Biofire® FilmArray® Pneumonia (PN) Panel (BioFire Diagnostics, Salt Lake City, UT, USA).

**Methods:**

A retrospective chart review was performed on all BAL specimens that had both a PN panel test and BR-PCR performed from January 2020 to May 2022 at all Indiana University affiliated hospitals. The PN panel test was performed in-house as per laboratory protocol, while BR-PCR was performed in a reference laboratory. Outcomes assessed included turn-around times (TAT), sensitivity and specificity of BR-PCR and clinical impact, if any.

**Results:**

A total of 68 BAL specimens from 53 patients were identified (83% of patients were immunocompromised). Percent positivity for the PN panel was 19% and that of BR-PCR was 18%. With the PN panel used as the gold standard, the sensitivity and specificity of BR-PCR was 85% and 98%, respectively. Only one respiratory organism was detected by BR-PCR but not by the PN panel, and it was not considered pathogenic or to have a significant clinical impact. The median TAT for the PN panel was 2.1 hours (1.8, 3.2) versus 7.8 days (6.9, 10.4) for BR-PCR.

**Conclusion:**

In our cohort of patients, BR-PCR testing was not superior to the Biofire® FilmArray® Pneumonia (PN) Panel when used to detect certain bacterial etiologies of pneumonia. Additionally, faster TAT for the panel test has the potential to enhance antimicrobial stewardship practices by enabling better antibiotic utilization. Adjunctive BR-PCR testing may be useful for clinical care when conventional testing is negative and patients are at risk for a variety of potential pathogens, including fungi.

**Disclosures:**

**All Authors**: No reported disclosures

